# Androgen receptor signaling organizes ferroptosis escape in castration-resistant prostate cancer: a threshold-based model for therapeutic timing

**DOI:** 10.3389/fonc.2026.1884640

**Published:** 2026-07-01

**Authors:** Canlin Wang, Jinting Lv, Jiahe Wu, Shuli Wang, Shuqi Song

**Affiliations:** Guang’anmen Hospital, China Academy of Chinese Medical Sciences, Beijing, China

**Keywords:** androgen receptor signaling, castration-resistant prostate cancer, ferroptosis escape, mitochondria, precision combination therapy, therapeutic resistance, vulnerability window

## Abstract

Castration-resistant prostate cancer (CRPC) remains the lethal endpoint of prostate cancer progression, driven by the persistence and adaptive reprogramming of androgen receptor (AR) signaling under therapeutic pressure. Although AR–ferroptosis crosstalk has been increasingly recognized, current models are largely descriptive and fail to explain why ferroptosis escape becomes a stable feature of resistant disease. Here, we propose that in CRPC, AR signaling functions not merely as a molecular regulator, but as the dominant state organizer of ferroptosis resistance, whereas ferroptosis influences AR primarily through a special feedback modulation rather than as an initiating driver of resistance evolution. We further suggest that this state can be understood through the concept of a ferroptosis threshold, which is shaped by AR across four interconnected axes: anti-ferroptotic defense, membrane lipid substrate ecology, edox buffering and iron toxicity boundary. Within this framework, mitochondria emerge not as passive effectors, but as central integrative hubs that couple metabolic rewiring, iron handling, reactive oxygen species control, and organelle communication to consolidate ferroptosis resistance. Building on this view, we propose a temporal model of AR-driven ferroptosis escape in CRPC, spanning an early vulnerability window after AR pathway inhibition, a phase of adaptive anti-ferroptotic reconstruction, and the eventual establishment of a resistant steady state. This model provides a theoretical basis for stage-specific therapeutic intervention. It supports both “early interception” during the vulnerability window and “late-state destabilization” after resistant homeostasis has been established. In addition, it provides a rationale for biomarker-guided dynamic monitoring and temporally informed combination therapeutic strategies.

## 1 Introduction

Prostate cancer has increasingly become a major public health challenge in men’s health worldwide. Globally, prostate cancer is the second most commonly diagnosed malignancy in men (1), and its disease burden is expected to continue rising, with the number of new cases projected to reach approximately 2.9 million by 2040 (2). For more than 80 years, the therapeutic landscape has centered on disrupting AR signaling. As a ligand-activated transcription factor, AR is a key driver of prostate cancer initiation and progression (3). On this basis, androgen deprivation therapy (ADT) was developed. Ithas long been regarded as the “gold standard” for the treatment of advanced prostate cancer. Although most prostate cancers are initially sensitive to ADT, sustained androgen-deprivation pressure inevitably drives disease evolution. In most patients with advanced disease, progression to CRPC occurs within 18 to 24 months after treatment, a stage that is usually accompanied by a marked deterioration in clinical outcome. It has been reported that the median overall survival thereafter is approximately 36 months (4, 5). Androgen receptor signaling inhibitors (ARSIs) have significantly improved survival outcomes in patients with prostate cancer; however, these benefits are often not durable, and most patients ultimately develop resistance and disease progression (6). The emergence of resistance is mainly associated with multiple mechanisms, including AR gene amplification, gain-of-function mutations,the generation of splice variants, and enhanced intratumoral androgen synthesis (7). Although this framework, centered on “AR reactivation,” remains necessary for explaining CRPC resistance, it is no longer sufficient to fully account for the metabolic adaptation, lipid remodeling, oxidative stress tolerance, and multidrug resistance that tumors acquire under therapeutic pressure (8, 9). In other words, resistance in CRPC is not merely a problem of restored receptor signaling, but also one of systemic remodeling across metabolic, membrane lipid, and redox networks.

Ferroptosis has been widely recognized as a potential therapeutic vulnerability in a variety of treatment-resistant tumors (10). CRPC is particularly well suited to be examined through the lens of ferroptosis. Its progression is accompanied by profound lipid metabolic remodeling, redox homeostasis rewiring, and enhanced stress adaptation. These factors jointly determine cellular sensitivity to ferroptosis (8–10). Notably, during the initial response phase, AR inhibition can promote ferroptosis in CRPC cells by inducing the accumulation of lipid peroxidation. In the subsequent adaptive or resistant phase, tumor cells can re-establish membrane lipid homeostasis and achieve ferroptosis escape through the maintenance of AR variants and metabolic reprogramming. This suggests that ferroptosis may serve as an indicator for assessing whether a resistant state is becoming stabilized and whether an actionable therapeutic window still exists (11).

**Figure 1:**
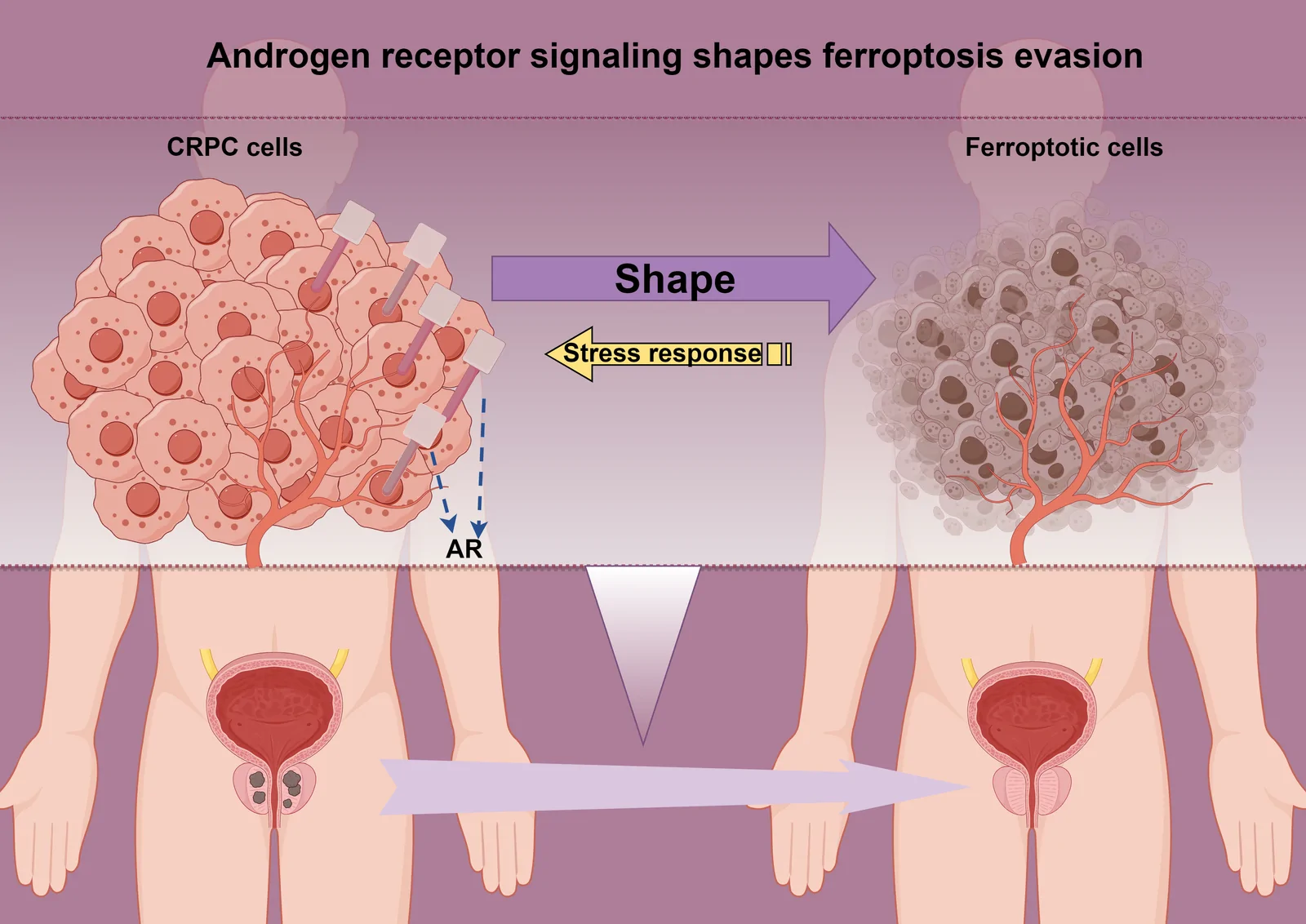
Androgen receptor signaling shapes ferroptosis evasion. AR signaling sets the ferroptosis threshold, whereas ferroptosis primarily modulates AR activity through stress responses. By Figdraw.

Current studies have largely focused on the bidirectional interplay between AR and ferroptosis, suggesting that AR inhibition may influence ferroptosis sensitivity, whereas ferroptosis-associated oxidative stress may in turn modulate AR activity (12). However, in the specific disease context of CRPC, the available evidence may better support a hierarchical organizational model in which AR signaling acts as the upstream architect of ferroptosis-related cellular states. The central issue in CRPC is therefore not simply whether ferroptosis occurs, but rather how AR systematically resets the ferroptosis threshold and how ferroptosis influences AR primarily through a special feedback modulation (10–12) (**Figure 1**). In this review, we therefore focus on AR signaling as the principal axis shaping ferroptosis escape in CRPC. Particular emphasis is placed on the pathways through which AR orchestrates ferroptosis escape in CRPC and on the key nodes that may constitute clinically actionable intervention windows. Through this discussion, we aim to integrate AR signaling, ferroptosis mechanisms, and stage-specific intervention strategies in CRPC into a unified model that can inform both mechanistic investigation and therapeutic design.

## 2 Literature search strategy and conceptual framework development

To develop the conceptual framework proposed in this review, a structured literature search was conducted using PubMed and Web of Science Core Collection from database inception through March 2026. The search strategy was designed to capture studies related to ferroptosis regulation in prostate cancer, with particular emphasis on AR signaling and mechanisms associated with ferroptosis resistance in CRPC. The primary search terms included combinations of “ferroptosis,” “androgen receptor,” “AR signaling,” “AR variants,” “AR-V7,” “castration-resistant prostate cancer,” “CRPC,” “SLC7A11,” “GPX4,” “glutathione,” “lipid peroxidation,” “polyunsaturated fatty acids,” “monounsaturated fatty acids,” “MBOAT2,” “iron metabolism,” “ferritinophagy,” “NCOA4,” “STAMP2,” “lactoferrin,” “mitochondria,” “mitochondria-associated endoplasmic reticulum membranes (MERCS),” and “oxidative stress.” Boolean operators (AND, OR) were used to combine search terms. Reference lists of key publications and relevant review articles were also manually screened to identify additional studies of potential relevance. Studies were considered eligible if they (i) investigated ferroptosis-related mechanisms in prostate cancer or CRPC, (ii) examined the role of AR signaling in cellular metabolism, redox homeostasis, lipid remodeling, iron regulation, or therapy resistance, or (iii) provided mechanistic, translational, or clinical evidence relevant to ferroptosis susceptibility. Original research articles, preclinical studies, and authoritative review articles were included. Studies lacking mechanistic relevance, focused exclusively on non-prostate malignancies without transferable mechanistic insights, or providing insufficient experimental evidence were excluded from the primary conceptual analysis.

This work employed a conceptual modeling approach to synthesize current evidence into an integrated mechanistic framework. Mechanisms were hierarchically prioritized according to four criteria: (i) their position within regulatory networks, particularly their capacity to function as upstream regulators; (ii) the breadth of their influence across multiple ferroptosis-related pathways; (iii) the strength, consistency, and reproducibility of supporting evidence across experimental systems; and (iv) their potential translational and therapeutic relevance. This evidence-informed framework forms the basis for our proposal that AR functions as the master orchestrator of ferroptosis escape in CRPC.

## 3 How AR signaling becomes the organizer of ferroptosis escape in CRPC

The development and progression of CRPC fundamentally occur under the sustained selective pressure imposed by long-term ADT and next-generation ARSIs (6, 7). Therefore, from the perspective of disease natural history and treatment dynamics, the first selective challenge faced by tumor cells is not whether ferroptosis occurs, but rather how survival and proliferation can be maintained under conditions of AR-axis suppression. This temporal sequence determines that changes in AR state generally precede changes in ferroptosis sensitivity.

A second reason is that AR possesses a cross-hierarchical capacity to organize cellular states. AR can serve as the dominant organizer of ferroptosis escape not only because it is a classical transcription factor, but also because in CRPC it exerts multi-layered state-shaping functions. Collectively, these changes influence whether lipid peroxidation remains a reversible stress response or progresses to lethal collapse. First, AR can directly regulate key nodes. Examples includetranscriptional control of cysteine utilization programs (e.g.,SLC7A11) and membrane phospholipid remodeling programs (e.g., MBOAT2). Through these mechanisms, AR simultaneously acting on both the antioxidant defense system and the membrane lipid substrate axis (11, 13). Second, AR signaling can indirectly reset the reductive infrastructure of tumor cells through metabolic reprogramming. This includes regulation of GSH/NADPH supply capacity (14), lipid synthesis and turnover pathways (13), as well as mitochondrial oxidative metabolism and stress-buffering capacity (15). Collectively, these changes influence whether lipid peroxidation remains a reversible stress response or progresses to lethal collapse. Third, AR also influences the speed at which cells respond to acute therapeutic stress through crosstalk between non-genomic signaling and stress-response pathways, such as PI3K/AKT and MAPK, making the restoration of anti-ferroptotic programs more dynamic and adaptive (16).

Finally, ferroptosis is more appropriately viewed as a state output, whereas what truly feeds back onto AR is sublethal oxidative-lipid stress (12). Terminal ferroptosis represents a cell death outcome; once the lethal threshold is crossed, cells lose the capacity to continue remodeling AR programs. What can act in reverse on AR is therefore mainly sublethal oxidative-lipid stress that has not yet reached the point of lethality, such as bufferable lipid peroxidation, ROS accumulation, and membrane damage signals. This is also the key reason why this review adopts “AR shapes ferroptosis escape” rather than “ferroptosis shapes AR” as its central framework: the former describes the upstream organizing logic underlying the formation of the resistant state, whereas the latter more often reflects feedback regulation occurring under non-lethal stress conditions. In other words, in CRPC, this sublethal state may enter a form of modulatory negative feedback.

### 3.1 What does this “modulatory negative feedback” actually mean?

Within an AR–ferroptosis coupling framework, describing the feedback simply as negative feedback is an oversimplification. Ferroptosis-related stress does not influence AR signaling in a single direction. In some settings, moderate, transient, and still-bufferable oxidative stress can enhance AR protein expression and thereby reinforce resistance (17). By contrast, when oxidative and lipid peroxidation stress becomes sustained or exceeds the buffering capacity of the cell, AR transcriptional activity is suppressed (18). For this reason, the more precise term is modulatory negative feedback.

**Figure 2:**
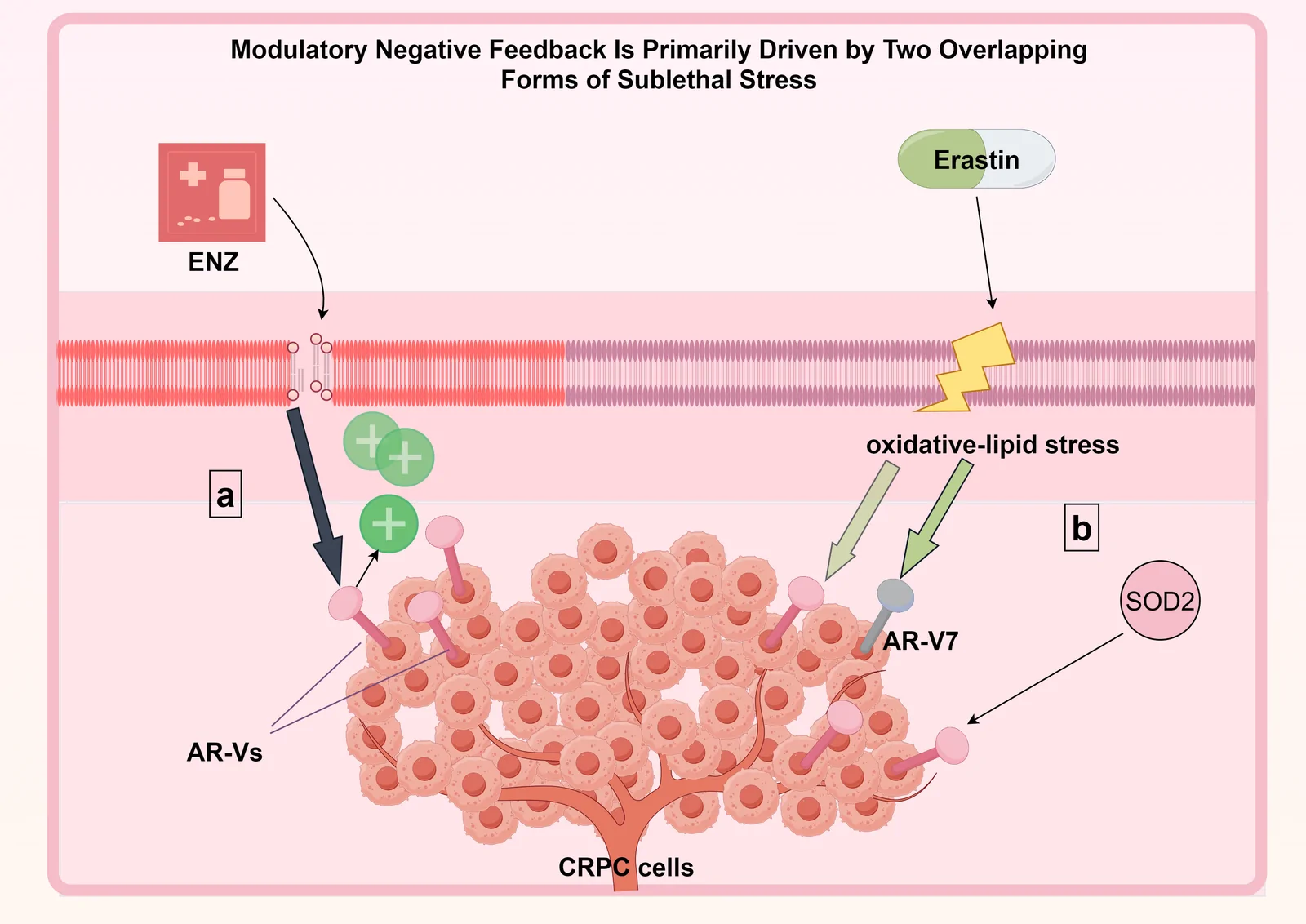
Modulatory Negative Feedback Is Primarily Driven by Two Overlapping Forms of Sublethal Stress. Two overlapping forms of sublethal stress may underlie the modulatory feedback of ferroptosis-related signals on AR output. **(a)** Ferroptotic stress denotes lipid peroxidation and membrane damage signals that remain below the lethal threshold. In enzalutamide-treated castration-resistant C4–2 cells, early membrane injury can arise before overt cell death, but this vulnerable state may later be offset by sustained AR-V-dependent restoration of SLC7A11 transcription, thereby promoting resistance. **(b)** Oxidative-lipid stress denotes a broader redox-imbalanced state linked to ROS accumulation and lipid peroxidation. In line with this, erastin suppresses AR-FL and AR-V expression, especially AR-V7, whereas SOD2 loss or inhibition enhances AR activity and AR-responsive transcription. By Figdraw.

This feedback is mainly driven by two overlapping forms of sublethal stress (12) (**Figure 2**). The first is ferroptotic stress, defined here as lipid peroxidation and membrane damage signals that have not yet crossed the lethal threshold.For example, in enzalutamide-treated castration-resistant C4–2 cells, glutathione depletion and loss of membrane integrity can emerge before overt cell death. These early changes may subsequently be counterbalanced by persistent AR-V-dependent restoration of SLC7A11 transcription, ultimately favoring resistance (**Figure 2a**). The second is oxidative-lipid stress, which includes reversible states such as ROS accumulation, phospholipid peroxidation, and redox disequilibrium (**Figure 2b**). Although these signals do not represent completed ferroptosis, they can be sensed as stress inputs by transcriptional networks, proteostasis systems, metabolic sensors, and organelle stress pathways, and in turn reshape AR output.Yang and colleagues (18) used GSH depletion and ROS accumulation as indicators of ferroptotic and showed that erastin markedly reduced AR-FL and AR-V expression, particularly AR-V7, in CRPC cells. By contrast, Sharifi and colleagues (19) demonstrated that loss or inhibition of the major mitochondrial antioxidant enzyme SOD2 increased AR activity and the expression of AR-responsive genes through a ROS-dependent mechanism. Collectively, these findings indicate that AR signaling can be influenced by ferroptosis-related stress, giving rise to a tunable feedback circuit.

### 3.2 Defining the ferroptosis threshold as a unifying mechanistic language

To move beyond linear, node-centric interpretations of the AR–ferroptosis relationship, we introduce the concept of the ferroptosis threshold. Here, the threshold does not refer to a fixed cutoff for any single molecule, but to the systems-level tipping point at which cells shift from bufferable lipid peroxidation to irreversible lethal membrane damage within a given spatiotemporal context (6, 7). This threshold is inherently dynamic, stage-dependent, and plastic, making it a more useful framework for capturing the combined effects of therapeutic pressure, AR rewiring, metabolic adaptation, and organelle coordination in CRPC. Conceptually, the ferroptosis threshold defines the functional “distance” between a tumor cell and lethal lipid peroxidation. When the threshold is high, cells can tolerate substantial peroxidative stress and remain viable; when it falls, damage that was previously manageable becomes fatal. Thus, the key question is not whether ROS rises or GPX4 is expressed in isolation, but how multiple regulatory modules collectively position the cell relative to this lethal boundary.

We propose that the ferroptosis threshold is governed by five interconnected modules. These include: (i)defense, representing antioxidant systems that directly suppress lethal lipid peroxidation;(ii)substrate, referring to the composition, distribution, and turnover of peroxidation-prone membrane lipids, particularly PUFA-containing phospholipids; (iii) redox buffering, representing the reducing infrastructure that maintains oxidative stress in a reversible range; (iv) iron, referring to the availability of catalytic iron and the control of its toxicity boundary; and (v) organelle networks, particularly mitochondria, together with the endoplasmic reticulum, especially MERCs, and peroxisomes, which spatially organize lipid remodeling, ROS propagation, iron handling, and damage amplification. In this sense, the organelle network does not simply add a fifth layer, but spatially integrates the other four (**Table 1**). Within CRPC, the AR–ferroptosis relationship is therefore better understood as an asymmetric coupling architecture. AR signaling functions as the dominant organizing axis, systematically resetting the ferroptosis threshold through five interconnected modules: defense, substrate, redox buffering, iron, and organelle-network gating. In contrast, sublethal ferroptotic stress operates mainly as a feedback-modulatory loop that reprograms AR output and shapes the tempo of resistant-state stabilization.

**Table 1:** AR-shaped multi-layer ferroptosis escape modules in CRPC.

Module	Key mediators	Anti-ferroptotic role	Tumor	Evidence Strength	References
Defence axis	SLC7A11	AR-V upregulates SLC7A11 to sustain GSH synthesis and GPX4-mediated peroxide detoxification.	LNCaP, 22Rv1, HEK293T and C4–2	Direct	(11)
	NEDD4L	SLC7A11 stabilization via reduced NEDD4L-mediated degradation.	C4–2 and 22RV1	Direct	(23)
	Imperatorin	Imperatorin suppresses SLC7A11/GPX4 and enhances ACSL4-linked lipid peroxidation.	WPMY-1, RWPE-1,DU145,PC3, C4–2 and C4-2R	Direct	(24)
	AR, SP1	AR–SP1 cooperatively promotes GPX4 transcription and peroxide detoxification.	LNCap95 and 22RV1	Indirect	(26)
	DHODH	DHODH reduces CoQ to CoQH2 in the inner mitochondrial membrane and suppresses ferroptosis in parallel with mitochondrial GPX4.	Non-prostate cancer models	Inferred(not directly regulated by AR)	(30)
	GCH1, BH4/BH2	GCH1–BH4/BH2 limits ferroptosis through lipid remodelling.	Non-prostate cancer models	Inferred(not directly regulated by AR)	(32)
Membrane lipid substrate ecology	NUS1, AR	NUS1 sustains AR abundance and signalling; its loss increases lipid peroxidation and ferroptosis sensitivity.	LNCaP、C4-2B、22Rv1、PC3、DU145 and MR-49F	Direct	(43)
	MBOAT2	AR upregulates MBOAT2 to reduce membrane combustibility.	HT1080, 293T, HPAC, MIA PaCa-2, PANC-1, Hs766T, Panc 05.04, Capan-1, 22Pc-EP, VCaP, MCF7, PC3, DU145	Indirect	(13)
	Darolutamide, SREBP1, FASN	AR blockade suppresses SREBP1–FASN, disrupts phospholipid balance, and favours peroxidation-prone states.	C4–2 and LNCaP	Direct	(45)
	ACSM1, ACSM3	AR-driven ACSM1/3 upregulation promotes tolerance to toxic medium-chain fatty acids and confers resistance to ferroptosis-inducing agents and AR antagonists.	LNCaP、22Rv1、VCaP、PC3、V16D and 49FENZR	Direct	(46)
Redox buffering	Trx	The Trx system buffers oxidative stress; its disruption by GA or CD-15 promotes ferroptotic cell death.	PCAP, B6WT, 1–4 PC3, LNCaP, DU145; PC-3, DU145	Inferred(Relationship with AR unclear)	(49, 50)
	Nrf2, Melatonin	Melatonin weakens AR-driven MCM5, disrupts MCM5–NRF2 coupling, and suppresses GPX4.	22Rv1 and C4‐2b	Direct	(51)
	Src, Raf, ERK, PI3K, Akt, AR	AR-associated rapid signalling redistributes survival and stress-response resources before full transcriptional rewiring.	LNCaP	Indirect	(53, 54)
	H2O2, AR, PSA	Low-dose H_2_O_2_ reactivates the AR–PSA axis.	LNCaP and 22RV1	Direct	(17)
Iron toxicity boundary	AR, LTFe, LTF	AR negatively regulates the LTFe–LTF axis, thereby constraining LIP expansion.	LNCaP	Direct	(59–61)
	STAMP2/STEAP4	AR-driven STAMP2/STEAP4 promotes Fe3+ reduction to Fe2+ while consuming NADPH.	LNCaP and VCaP	Indirect	(62, 63)
	NCOA4	Androgen signalling promotes NCOA4-dependent ferritinophagy and expands LIP.	LNCaP, LAPC4, HEK293T, and NK92	Indirect	(64)
Mitochondrial execution hub	ENZ	ENZ-treated prostate cancer cells show ferroptosis-associated mitochondrial shrinkage, and increased membrane density.	LNCaP, 22Rv1, HEK293T and C4–2	Direct	(11)
	DECR1	DECR1 downregulation enhances mitochondrial lipid peroxidation and promotes ferroptosis.	LNCaP,VCaP, 22RV1, V16D MR49F	Direct	(65)
	MPC	AR drives transcriptional dependence on MPC-mediated pyruvate import, thereby sustaining TCA output, lipid synthesis and oxidative phosphorylation.	RWPE1,22RV1,LNCaP,DU145, PC3, LAPC4,ABL, LuCaP58, LuCaP78 PDX,LuCaP35CR,LuCaP78,VCaP, C4-2	Indirect	(66)

## 4 How AR orchestrates ferroptosis escape

### 4.1 AR shapes the anti-ferroptotic defense axis

**Figure 3:**
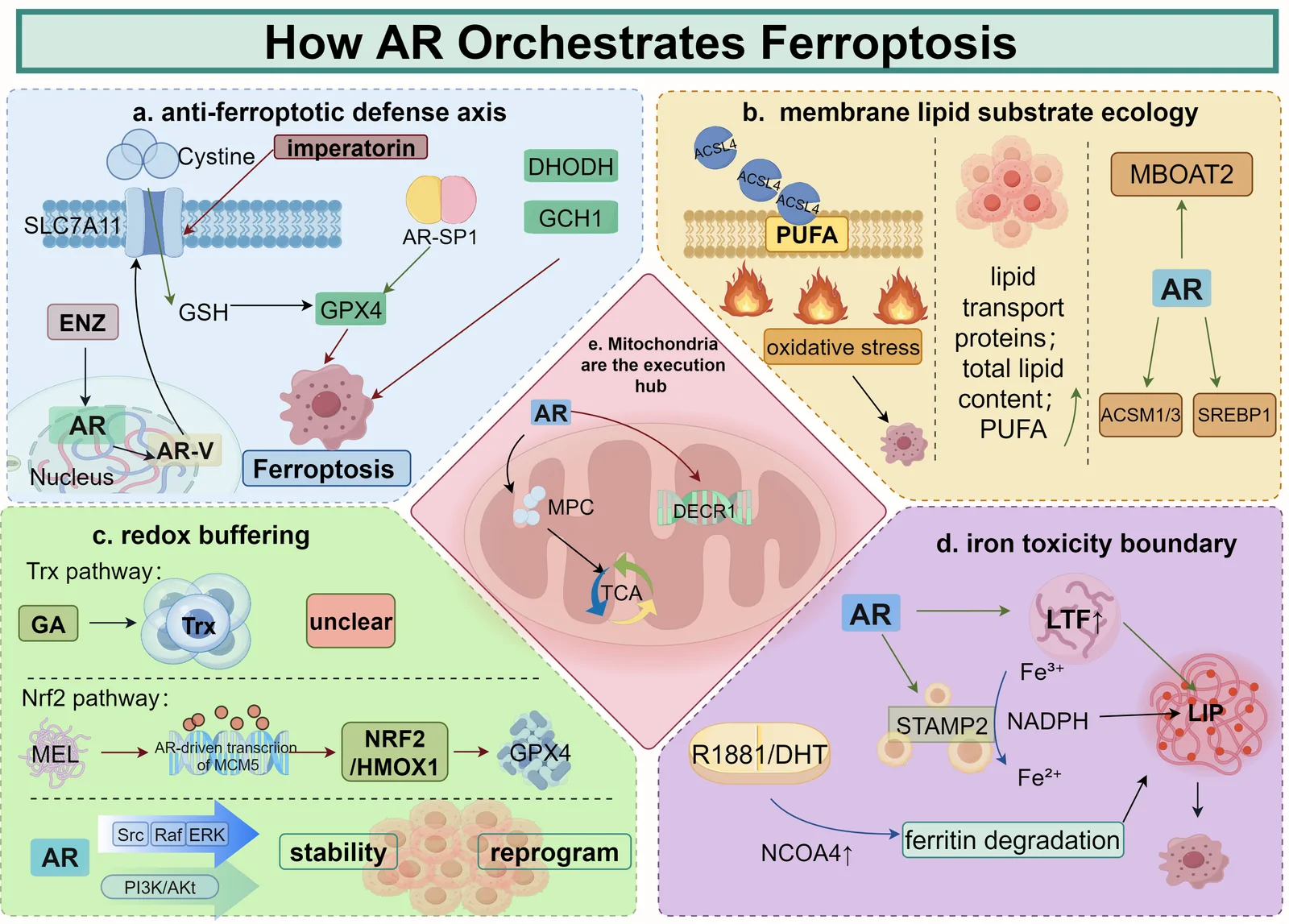
How AR Orchestrates Ferroptosis. **(a)** In CRPC cells, AR variants (AR-Vs) directly transcriptionally upregulate SLC7A11, thereby increasing cystine utilization, sustaining GSH synthesis, and reinforcing the anti-ferroptotic barrier. Imperatorin induces ferroptosis in prostate cancer cells by downregulating SLC7A11/GPX4, enhancing ACSL4-mediated lipid peroxidation.AR can also cooperate with SP1 to form a transcriptional complex that promotes GPX4 expression, further suppressing ferroptosis. In parallel, DHODH and GCH1 also function as ferroptosis suppressors. **(b)**. Oxidative stress can be conceptualized as the ignition source, whereas PUFA-containing phospholipids constitute the fuel layer that determines whether ferroptosis can be initiated and propagated. ACSL4, a key member of the long-chain acyl-CoA synthetase family, drives membrane lipid remodeling by enriching long-chain ω6 PUFAs and thereby governs cellular susceptibility to ferroptosis. In CRPC cells, multiple lipid transport proteins are upregulated, accompanied by increased total lipid content and a particularly marked accumulation of PUFAs. Within this context, AR reshapes membrane lipid composition and ferroptotic susceptibility through several coordinated mechanisms: it upregulates MBOAT2 to reduce membrane combustibility, enhances SREBP1-driven lipogenic programs to preserve phospholipid homeostasis, and induces ACSM1/3 expression to support lipid metabolic adaptation and ferroptosis resistance. **(c)** At the level of Trx system, GA disrupts redox homeostasis in advanced prostate cancer cells, thereby promoting ferroptosis. Although the Trx system appears to have considerable relevance to ferroptosis regulation, its specific crosstalk with AR remains unclear and warrants further investigation. At the level of the NRF2 pathway, MEL suppresses AR-driven transcription of MCM5. Reduced MCM5 in turn leads to dysregulated activation of the NRF2/HMOX1 axis, suppression of GPX4, and accumulation of ferroptotic markers. In parallel, AR can also initiate rapid stress-adaptive signaling, including fast activation of the Src/Raf/ERK pathway and parallel or cooperative activation of the PI3K/Akt pathway, thereby endowing prostate cancer cells with a “stabilize first, then reprogram” capacity under therapeutic stress. **(d)** In CRPC, AR shapes the iron-toxicity boundary by negatively regulating LTF, thereby influencing intracellular iron availability and ferroptotic susceptibility. Increased LTF expression expands the LIP and promotes a ferroptosis-permissive state by enhancing lipid peroxide generation. In parallel, AR also regulates STAMP2, an androgen-inducible metalloreductase that promotes the reduction of Fe³^+^ to Fe²^+^ while simultaneously consuming NADPH, thereby increasing intracellular ROS levels. In addition, R1881/DHT promotes ferritin degradation through upregulation of NCOA4 and enhanced NCOA4–ferritin interaction, further facilitating iron release from storage pools. **(e)** AR can influence ferroptosis through mitochondrial metabolic regulators. Downregulation of DECR1, a negatively regulated AR target gene, increases mitochondrial oxidative stress, particularly lipid peroxidation, thereby promoting ferroptosis. In parallel, AR also establishes a transcriptional dependence of prostate cancer cells on MPC-mediated pyruvate import, and inhibition of MPC correspondingly suppresses TCA cycle output, highlighting the importance of AR-controlled mitochondrial metabolism in shaping ferroptotic vulnerability. By Figdraw.

In CRPC, AR signaling establishes a cellular defense system against ferroptosis by regulating the SLC7A11/GSH/GPX4 axis and its associated bypass pathways (**Figure 3a**). As a key upstream node in this axis, SLC7A11 controls intracellular GSH synthesis and thereby influences the activity of GPX4, a core antioxidant enzyme that suppresses lipid peroxidation, ultimately determining cellular sensitivity to ferroptosis (20, 21). In CRPC cells, AR-V can directly transcriptionally regulate SLC7A11, supplying sufficient substrate for GSH synthesis, and further strengthening this anti-ferroptotic barrier (11).

Enzalutamide (Enz), a potent inhibitor of AR signaling, suppresses CRPC cell proliferation primarily by blocking AR nuclear translocation, preventing its binding to androgen response elements, and impairing coactivator recruitment (22). Enz can induce the accumulation of lipid peroxides by reducing intracellular GSH levels; however, AR-V-mediated upregulation of SLC7A11 can restore GSH synthesis and supply, rebuild the antioxidant defense system, and thereby block ferroptotic progression, representing one of the key mechanisms underlying Enz resistance in CRPC cells (11). Further studies suggest that AR-mediated transcriptional activation and NEDD4L-dependent stabilization may represent sequential yet complementary regulatory events converging on SLC7A11 accumulation. In the proposed model, reactivated AR/AR-V signaling first restores SLC7A11 expression at the transcriptional level, whereas suppression of NEDD4L-mediated ubiquitin-dependent degradation under sustained Enz-selective pressure subsequently prolongs SLC7A11 protein stability and promotes its persistent accumulation (23). Through the coordinated action of these transcriptional and post-translational mechanisms, CRPC cells progressively rebuild their antioxidant defense system, reinforce ferroptosis resistance, and ultimately acquire a stable Enz-resistant phenotype.

Notably, the bioactive natural compound imperatorin has shown synergistic effects with Enz *in vitro*, further supporting the central role of the SLC7A11/GPX4 axis as an anti-ferroptotic defense mechanism in resistance formation and highlighting its potential as a target for combination therapy. Experimental evidence indicates that imperatorin suppresses prostate cancer cell proliferation, migration, and invasion in a dose-dependent manner by downregulating SLC7A11/GPX4, upregulating ACSL4-mediated lipid peroxidation, and ultimately triggering ferroptosis (24). In addition, the transcription factor specificity protein 1 (SP1) is frequently aberrantly activated in tumor tissues and is involved in the regulation of tumor proliferation, angiogenesis, and metastasis (25). Studies have shown that AR can also form a transcriptional complex with SP1 to cooperatively promote GPX4 transcription, thereby suppressing ferroptosis (26). Meanwhile, PHGDH, a metabolic node associated with Enz resistance, can help maintain this defense axis by sustaining redox homeostasis and GSH levels (27). Together, these regulatory mechanisms not only underscore the central role of AR signaling in setting the ferroptosis threshold, but also reveal a more complex dynamic relationship between AR and ferroptosis: AR signaling does not simply “regulate” ferroptosis, but dynamically tunes ferroptotic sensitivity and the rate at which antioxidant capacity is restored under therapeutic pressure.

Finally, CRPC may also reinforce its ferroptosis defense through compensatory bypass pathways. FSP1, a key component of the non-mitochondrial coenzyme Q antioxidant system, is regulated by nuclear AR protein in a manner that appears independent of its canonical transcriptional activity, and acts in parallel with the GPX4 pathway to suppress intracellular lipid peroxidation (28, 29). DHODH, another ferroptosis-suppressive enzyme, functions in parallel with mitochondrial GPX4 and inhibits ferroptosis in the inner mitochondrial membrane by promoting the reduction of CoQ to CoQH_2_ (30). Notably, subsequent studies have suggested that at high concentrations, DHODH inhibitors may also exert off-target inhibition of FSP1 (31). Therefore the attribution of pharmacological sensitization effects should be interpreted cautiously in light of dosage and genetic evidence. In addition, GTP cyclohydrolase 1(GCH1) and its metabolic derivatives tetrahydrobiopterin/dihydrobiopterin (BH4/BH2) also function as ferroptosis suppressors; in GCH1-expressing cells, BH4/BH2 synthesis drives lipid remodeling and thereby inhibits ferroptosis (32). Through the coordinated action of these bypass pathways, CRPC cells are often able to evade the failure of single-node inhibition.

### 4.2 AR shapes membrane lipid substrate ecology

One of the key determinants of ferroptosis is whether the plasma membrane contains a sufficient amount and an appropriate distribution of peroxidation-susceptible substrates (**Figure 3b**). Polyunsaturated fatty acids (PUFAs) within phospholipids, such as arachidonic acid (AA) and adrenic acid (AdA), undergo peroxidation in the presence of reactive oxygen species (ROS), thereby strongly promoting ferroptotic cell death (33). Oxidative stress can be viewed as the “ignition source,” whereas PUFA-containing phospholipids, particularly those enriched in AA and AdA, serve as the “fuel layer” that determines whether ferroptosis can be initiated and propagated. Oxidized AA/AdA-phosphatidylethanolamines (PEs) have been identified in breast cancer cells as lethal lipid signals that lie closer to the execution layer of ferroptosis (34). ACSL4, a member of the long-chain acyl-CoA synthetase family, plays a central role in membrane lipid remodeling by enriching long-chain ω6 PUFAs and thereby determining cellular susceptibility to ferroptosis (34). Importantly, however, not only the total PUFA abundance but also the specific phospholipid pools into which PUFAs are incorporated is critical for ferroptosis (35).Thus, the ability of CRPC cells to remain viable despite substantial oxidative stress may, at least in part, reflect the fact that the “fuel” has been redistributed.

Disruption of lipid homeostasis is one of the defining metabolic features of the transition from prostate cancer to CRPC (36). In CRPC cells, the expression of multiple lipid transport proteins is increased, total lipid content rises, and PUFA levels in particular are significantly elevated (37). In addition, androgens further increase lipid abundance in prostate cancer cells by enhancing lipid synthesis and uptake, whereas AR antagonists suppress these lipid-supply pathways (38–40). In prostate cancer, high PUFA levels can also increase membrane permeability and migratory capacity, thereby facilitating membrane-associated biological processes such as vesicle formation, membrane fusion, cell-cell interactions, and the regulation of membrane enzymes, receptors, and transporters (41). Increased membrane PUFA content not only enhances membrane fluidity and lipid peroxidation, but also increases vulnerability to glutathione peroxidase 4 (GPX4) inhibition, thereby promoting ferroptosis susceptibility (42).

AR signaling can shape the membrane lipid environment relevant to ferroptosis through lipid metabolic reprogramming. The lipid metabolic checkpoint NUS1 plays an important role in maintaining AR protein levels and signaling activity, and loss of NUS1 increases lipid peroxidation and sensitizes cells to ferroptosis (43). In addition, MBOAT2, a membrane lipid-remodeling factor regulated by AR, has also been identified as a ferroptosis-suppressive gene. MBOAT2 selectively transfers monounsaturated fatty acids (MUFAs) into lyso-phosphatidylethanolamine (lyso-PE), thereby increasing cellular PE-MUFA and correspondingly decreasing cellular PE-PUFA levels, and MBOAT2 can be directly upregulated by AR. Mechanistically, MBOAT2/LPCAT4 functions as a lysophospholipid acyltransferase involved in phospholipid remodeling. Earlier enzymatic studies demonstrated substrate preferences toward oleoyl-CoA and ethanolamine-containing lysophospholipid acceptors, supporting its capacity to enrich MUFA-containing PE species (44). Consistently, lipidomic analyses have shown that MBOAT2 increases PE-MUFA while reducing ferroptosis-prone PE-PUFA species, thereby shifting membrane lipid composition toward a less peroxidizable state. Although the precise structural determinants governing MBOAT2 substrate recognition remain incompletely understood, these findings support a model in which AR-driven MBOAT2 expression promotes ferroptosis resistance through selective membrane lipid remodeling. Anti-AR agents such as enzalutamide and ARV-110 sensitize AR-positive prostate cancer cells to ferroptosis by downregulating MBOAT2 expression, indicating that AR can reshape the configuration and distribution of “combustible” membrane lipids (13).Darolutamide, another AR inhibitor, can promote ferroptosis by downregulating SREBP1, which then inhibits the transcription of FASN. FASN knockdown modulates phospholipid remodeling by disrupting the balance between PUFAs and saturated fatty acids (SFAs), which induces ferroptosis (45). AR-regulated acyl-CoA synthetase medium-chain family members 1 and 3 (ACSM1 and ACSM3), which function as major regulators of the prostate cancer lipidome, enhance energy production through fatty acid oxidation. Studies have shown that metabolic dysregulation caused by ACSM1/3 loss can trigger ferroptosis, whereas elevated ACSM1/3 activity enables prostate cancer cells to tolerate toxic levels of medium-chain fatty acids and promotes resistance to both ferroptosis-inducing agents and AR antagonists (46). Thus, at the substrate level, AR acts not only by modulating “membrane lipid combustibility,” but also by regulating “fuel supply” through fatty acid synthesis and lipid provisioning networks, together determining the substrate-side height of the ferroptosis threshold.

More broadly, prostate cancer cells may also sequester PUFAs into lipid droplets to avoid crossing the ferroptosis threshold. AR-targeted therapies, including enzalutamide, can induce marked lipid uptake and membrane lipid remodeling in prostate cancer cells, thereby generating a treatment-tolerant transitional state that is more sensitive to GPX4 inhibition, yet also highly plastic (42). In more general resistance models, cell-cycle arrest or slow-cycling states can promote DGAT-dependent lipid droplet formation, thereby sequestering PUFAs as triacylglycerols (TAGs), lowering membrane phospholipid peroxidation, and suppressing ferroptosis (47). Conversely, lipophagy can release these sequestered lipid substrates and promote ferroptosis (48). Under therapeutic pressure, CRPC may therefore continuously rewrite the fate of PUFAs: whether they are incorporated into membranes or stored in lipid droplets, and whether they are exposed as combustible substrates or temporarily isolated as “reserve fuel.” Taken together, current studies highlight the central role of AR in shaping membrane lipid substrate ecology. However, how AR regulates the distribution of lipid substrates across distinct phospholipid pools remains unclear. In addition, how to precisely control lipid droplet formation and degradation in order to prevent therapeutic resistance in CRPC represents an important direction for future investigation.

### 4.3 AR shapes redox buffering

Ferroptosis is not a linear event that is automatically triggered whenever ROS levels rise; rather, the critical issue is whether these oxidative “sparks” can be continuously amplified at the membrane lipid level and ultimately exceed the cellular capacity for detoxification and repair (33) (**Figure 3c**). Increased PHGDH expression can suppress ferroptosis by maintaining redox homeostasis in enzalutamide-resistant CRPC cells (27). At the level of the thioredoxin (Trx) system, gambogic acid (GA) disrupts redox homeostasis in advanced prostate cancer cells, leading to elevated ROS levels and thereby inducing both apoptosis and ferroptosis. Mechanistically, GA mainly acts by inhibiting thioredoxin, a key component required for cellular antioxidant defense and protein-reducing activity (49). Compound CD-15, a chalcone derivative, has shown a better safety profile than the widely used clinical drug docetaxel; mechanistically, CD-15 is able to induce ferroptosis in a TrxR-dependent manner (50). Although the Trx system shows considerable potential in ferroptosis regulation, its specific interaction with AR remains unclear and may represent an important direction for future investigation. At the level of the Nrf2 pathway, melatonin (MEL) weakens the liquid-liquid phase separation (LLPS) dynamics of AR and reduces AR-driven transcription of minichromosome maintenance protein 5 (MCM5). MCM5 is a clinical biomarker associated with aggressive prostate cancer and poor survival. Importantly, reduced MCM5 weakens its physical interaction with NRF2, leading to dysregulated activation of the NRF2/HMOX1 pathway, suppression of GPX4, and accumulation of ferroptotic markers (51). More broadly, cells can also compartmentally “truncate” lipid peroxidation chain reactions through the FSP1 system, which acts in parallel with GPX4, and the DHODH system in the inner mitochondrial membrane (29–31). This means that the same oxidative pressure may drive entirely different outcomes in different cells: some cells are ignited, whereas others are restrained by buffering systems and remain in a prolonged state of “high pressure without crossing the threshold.” In essence, the amplification or suppression of ferroptosis is a multi-compartment, spatially coupled process, rather than simply a function of total ROS intensity (52).

In addition, AR-mediated shaping of the redox axis does not rely exclusively on slow transcriptional reprogramming. Studies have shown that AR can trigger rapid signaling events, such as fast activation of the Src/Raf/ERK pathway and parallel or cooperative activation of the PI3K/Akt pathway. These non-genomic or quasi-non-genomic events provide cells with a stress-adaptive capacity of “stabilize first, then reprogram” (53, 54). This may help explain why both clinical observations and experimental models often reveal a transient window of ferroptotic vulnerability that is then rapidly “refilled” and closed: before the AR-V-driven transcriptional program is fully established, AR-associated rapid signaling may already begin to redistribute survival and stress-response resources (53, 54).

At the same time, oxidative stress does not always weaken AR signaling. Studies have shown that low-dose H_2_O_2_ can activate the AR–PSA axis, while USP36 can bind to AR under oxidative stress conditions and stabilize it through deubiquitination; knockdown of USP36 abolishes this H_2_O_2_-induced AR reactivation (17). This suggests that sublethal oxidative stress, under specific intensities and temporal contexts, may instead help AR reoccupy a dominant regulatory position. Most importantly, the redox axis defines two parameters that are highly relevant to therapeutic timing: window length and refill speed. The former determines how long cells remain ferroptosis-sensitive after AR inhibition, whereas the latter determines how quickly the resistant steady state is rebuilt.

### 4.4 AR shapes the iron toxicity boundary

Iron can be understood as the driving arm that determines whether the system will lose control (**Figure 3d**). Tumors with active androgen signaling may be intrinsically sensitive to iron, a feature that is also relevant to castration-resistant tumors in which AR signaling remains active. Combining iron with antiandrogen agents can induce ferroptosis in castration-resistant tumors in which androgen signaling is inactivated or absent (55). In ferroptosis, what truly determines whether chain lipid peroxidation can be continuously amplified is the cell’s ability to sustain a sufficient supply of reactive iron (10). From this perspective, the decisive factor is not the total iron content at a given moment, but rather the labile iron pool (LIP) and its dynamic replenishment capacity—namely, whether the combined “iron flux” generated by iron import (e.g., TfR1-related processes), iron storage/release (e.g., ferritinophagy), and iron efflux (e.g., FPN1 or ferritin export) is sufficient to sustain the lipid peroxidation chain reaction (56–58).

In CRPC, AR can negatively regulate the LTFe–LTF axis, thereby shaping the iron toxicity boundary. The LTF gene encodes lactoferrin, a well-recognized protein involved in iron transport and storage that is essential for maintaining cellular iron homeostasis. Increased LTF expression expands the labile iron pool and creates a cellular environment permissive for ferroptosis by enhancing lipid peroxide generation (59). LTFe promotes ferroptosis through upregulation of LTF (59–61). This regulatory mechanism enables AR to influence the threshold of iron-associated toxicity in CRPC cells through fine control of intracellular LIP. Another important AR-regulated factor is STEAP4 (with STAMP2 representing an alternative designation in a different expression context) (62). Androgens can increase STAMP2 expression, thereby promoting the reduction of Fe³^+^ to Fe²^+^ while simultaneously consuming available NADPH, increasing intracellular ROS levels and ultimately contributing to prostate cancer growth and progression (63). The significance of AR-mediated STAMP2 upregulation therefore lies not simply in increasing the expression of an iron metabolic protein, but in simultaneously driving Fe²^+^ generation and NADPH consumption. In effect, this places pressure on both sides of the ferroptosis threshold: the former increases the reactivity of radical-generating substrates, whereas the latter weakens the buffering capacity for detoxifying lipid peroxidation.

In prostate cancer, another key pathway that can push cells into the iron toxicity window is ferritinophagy. By activating NCOA4-mediated ferritin degradation, ferritinophagy expands the labile iron pool, promotes lipid peroxidation, and ultimately induces ferroptosis (64). Studies have shown that under androgen stimulation (R1881/DHT), prostate cancer cells undergo ferritin degradation accompanied by NCOA4 upregulation and enhanced interaction between NCOA4 and ferritin; correspondingly, inhibition of NCOA4 markedly attenuates androgen-induced ferritin degradation (64). These findings suggest that androgen signaling can mobilize intracellular iron stores, thereby remodeling iron homeostasis and increasing the propensity for iron-dependent damage. When this pathway is considered together with the AR–STAMP2/STEAP4 axis, a more explanatory model emerges: AR/androgen signaling not only promotes the reduction of Fe³^+^ to Fe²^+^ and consumes NADPH through STAMP2/STEAP4, but can also release stored iron from ferritin through NCOA4-dependent ferritinophagy. Acting in concert, these two pathways both expand the reactive iron pool and weaken the cell’s reductive buffering capacity, thereby increasing the efficiency with which lipid peroxidation chain reactions are amplified. More broadly, some tumors may retain a strong antioxidant defense yet simultaneously exist in a state of high iron retention or high iron release; once the defense barrier is breached, such cells may cross the ferroptosis threshold rapidly. By contrast, other tumors may confine damage to a prolonged sublethal range by enhancing iron efflux or limiting the effective LIP.

Whether LTF-, STAMP2/STEAP4-, and NCOA4-dependent pathways are co-activated within the same molecular subtype of CRPC remains unclear. Current evidence does not directly demonstrate coordinated upregulation of these pathways in a defined CRPC subtype. Nevertheless, these pathways regulate distinct yet interconnected aspects of iron metabolism: LTF contributes to iron uptake and redistribution, STAMP2/STEAP4 promotes ferric iron reduction and redox stress, whereas NCOA4-mediated ferritinophagy mobilizes stored iron and expands the LIP. Therefore, rather than functioning as redundant mechanisms, they are more likely to act as complementary regulatory modules that converge on expansion of the reactive iron pool and amplification of iron-dependent lipid peroxidation, thereby influencing the iron toxicity threshold and ferroptosis susceptibility of CRPC cells. Notably, available evidence suggests that STAMP2/STEAP4 and NCOA4 are preferentially associated with AR-active states, whereas the expression pattern of LTF across distinct CRPC subtypes and its relationship with AR signaling remain to be further elucidated.

### 4.5 Mitochondria are the execution hub through which AR shapes ferroptosis escape

If the ferroptosis threshold represents the master switch by which CRPC cells maintain survival under therapeutic pressure, then mitochondria function more like the executive hub of this switch, because they simultaneously integrate three decisive processes: metabolic flux, redox burden, and inter-organelle damage propagation (**Figure 3e**). Prostate cancer cells treated with ENZ exhibit mitochondrial shrinkage and increased membrane density, both characteristic features of ferroptosis (11). On the one hand, at the metabolic level, AR can influence ferroptosis through mitochondrial metabolic regulators. Downregulation of ACSM1/3 impairs mitochondrial function and induces oxidative stress and ferroptosis (46), and downregulation of DECR1 (a negatively-regulated AR target gene)also increases mitochondrial oxidative stress, particularly lipid peroxidation, and thereby triggers ferroptosis (65). Studies have further shown that AR drives a clear transcriptional dependence of prostate cancer cells on mitochondrial pyruvate carrier (MPC)-mediated pyruvate import, and that MPC inhibition concurrently suppresses TCA cycle output, lipid synthesis, and oxidative phosphorylation while activating the integrated stress response. These findings indicate that mitochondria are not merely a metabolic backdrop, but a central interface through which AR sustains tumor adaptation (66).

On the other hand, mitochondria themselves harbor multiple threshold nodes that directly influence ferroptosis outcome, including the DHODH-mediated mitochondrial CoQ defense system, NFS1-dependent Fe–S cluster assembly protection, and FXN-related mitochondrial iron homeostasis and Fe–S cluster maturation programs (67). More importantly, studies in breast cancer have localized the early spatial origin of ferroptotic membrane phospholipid peroxidation to ER–mitochondria contact sites (EMCSs/MERCS) and further shown that damage can spread from these contact sites to mitochondria, amplifying mitochondrial ROS production and mitochondrial fission (52). This suggests that, in the context of CRPC, treating mitochondria as merely one module among many would underestimate their role, because they in fact participate simultaneously in the generation, amplification, and gating of the ferroptosis threshold.

## 5 Temporal model: AR-driven ferroptosis escape is not an instantaneous event, but an evolvable state machine

**Figure 4:**
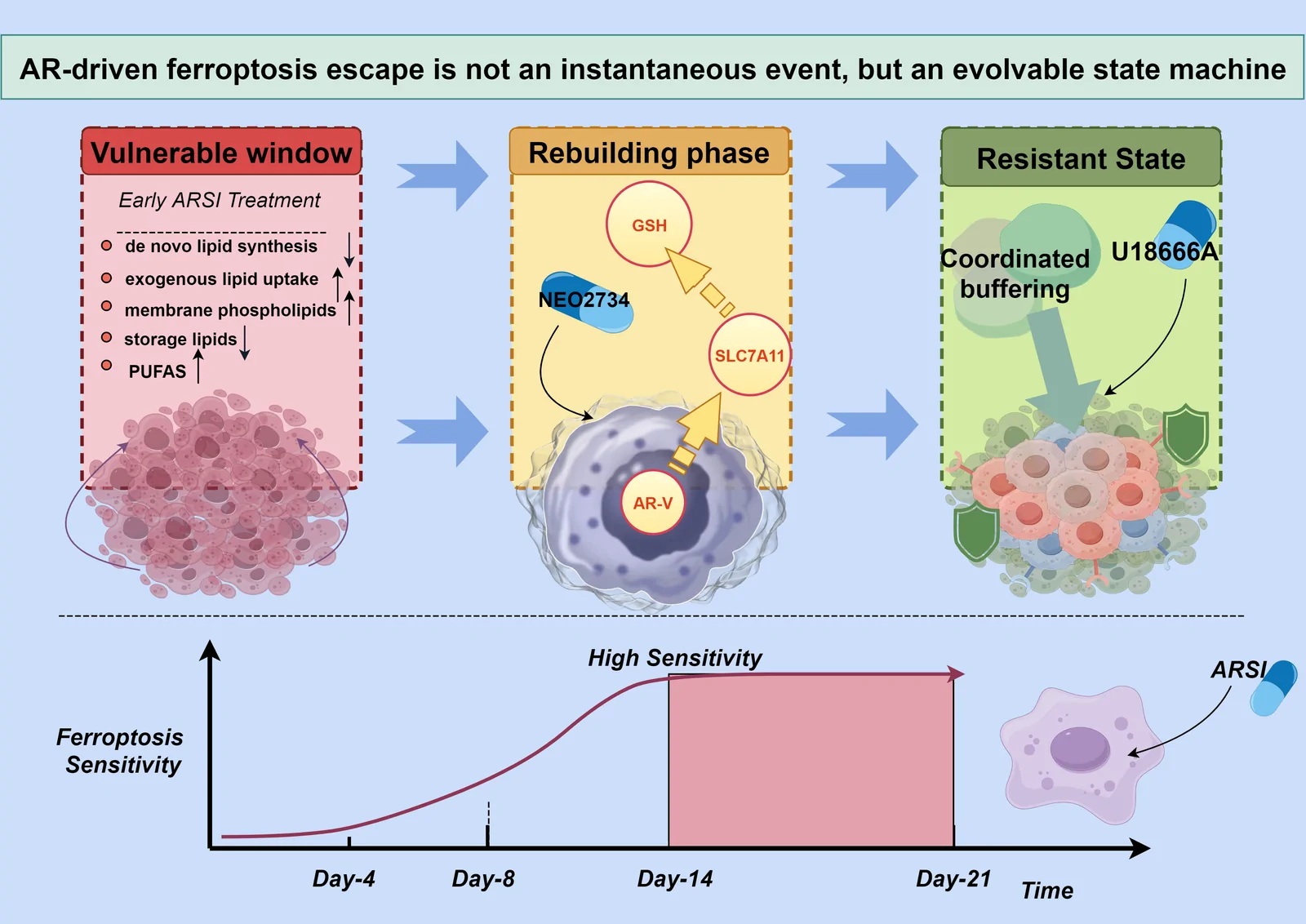
AR-driven ferroptosis escape is not an instantaneous event, but an evolvable state machine. Enzalutamide exposure drives a dynamic ferroptosis adaptation program in prostate cancer. Early treatment induces lipidomic remodeling, including reduced *de novo* lipogenesis, increased lipid uptake, membrane phospholipid accumulation, storage lipid depletion, and progressive PUFA enrichment, thereby creating a ferroptosis-hypersensitive state. ferroptosis sensitivity changes dynamically over the same treatment course, increasing at days 4–8, reaching a markedly hypersensitive state by day 14, and remaining elevated at day 21. Subsequently, AR variants restore SLC7A11 expression and GSH metabolism to promote ferroptosis escape, whereas NEO2734 can counter this process by suppressing AR-V expression. In the consolidated resistant state,survival depends on coordinated buffering across multiple adaptive modules rather than a single defense pathway, while enzalutamide-tolerant cells may remain vulnerable to U18666A. By Figdraw.

In CRPC, the relationship between AR signaling and ferroptosis is better understood as a state trajectory that is continuously rewritten under therapeutic pressure, rather than as a one-time upregulation or downregulation of a single molecule. At the onset of antiandrogen therapy, cells may first exhibit a vulnerable phenotype characterized by increased lipid peroxidation and imbalance in GSH supply. This is followed by the gradual restoration of AR variants, SLC7A11, and metabolic support networks, which drives cells from a state of high pressure without crossing the threshold into one of sustainable tolerance. Ultimately, multi-module compensation—including reinforcement of defense systems, remodeling of substrate availability, redox buffering, and organelle coordination—becomes consolidated into a stable resistant state (**Figure 4**) (**Table 2**). Due to the lack of comprehensive longitudinal studies, we integrated findings from multiple independent investigations to propose a temporal model describing how AR signaling regulates ferroptosis in CRPC. The present model should be regarded as most applicable to AR-dependent and AR-plastic CRPC states, whereas its applicability to AR-low, AR-independent, or neuroendocrine-like disease states remains to be determined.

**Table 2:** A three-stage model of ferroptosis remodelling during ARSI treatment in CRPC.

Stage	Biological state	Ferroptosis status	Key determinants	Therapeutic implication	References
Stage 1 | Pro-ferroptotic vulnerability window	AR output is rapidly suppressed, whereas adaptive rescue remains incomplete.	Lowered ferroptosis threshold; increased GPX4 dependence; heightened liability to lipid peroxidation.	ARSI induces a quiescent-like state with reduced proliferation, ATP production and mitochondrial activity. In parallel, therapy-driven lipid remodelling increases membrane susceptibility, including enhanced exogenous lipid uptake, membrane phospholipid accumulation, depletion of storage lipids, and progressive enrichment of PUFA-containing phospholipids. Ferroptosis hypersensitivity emerges early and becomes pronounced during days 14–21. In LNCaP and C4-2B cells, Enz is combined with non-toxic doses of lipase inhibitors (orlistat or CAY10499) or the fatty acid δ6 desaturase inhibitor SC26196 significantly attenuate RSL3-induced ferroptosis hypersensitivity.	Defines a time-sensitive interception window for combining ARSI with increasing lipase activity and fatty acid δ6 before adaptive rescue is established.	(42, 68, 69)
Stage 2 | AR-shaped rebuilding phase	AR-dependent adaptive programmes begin to restore anti-ferroptotic capacity.	Threshold re-elevation in progress; ferroptosis sensitivity is progressively restrained but not yet fully stabilized.	AR-V re-engages regulatory regions of SLC7A11, restoring GSH supply. ENZ may further enhance SLC7A11 stability through a NEDD4L-related mechanism. Dual inhibition with NEO2734 plus enzalutamide suppresses AR-variant expression, reduces SLC7A11 and increases 4-HNE, indicating that this rebuilding process remains pharmacologically disruptable.	This phase remains both delayable and destabilizable, and may therefore be particularly amenable to combinatorial strategies that simultaneously target AR plasticity and antioxidant rebuilding, such as dual inhibition with NEO2734 and enzalutamide.	(11, 23)
Stage 3 | Steady-state consolidation	A coordinated anti-ferroptotic resistant state is established through multilayer buffering and cooperative adaptation.	Re-elevated ferroptosis threshold; single-node ferroptosis induction is less durable.	Multiple modules begin to operate in a coordinated buffering manner, more effectively confining damage within a sublethal range.Adaptive dependence on lipid and cholesterol trafficking also increases, including NPC1/NPC2-linked pathways; accordingly, enzalutamide-tolerant cells display increased sensitivity to U18666A.	Supports combination regimens designed to dismantle the resistant steady state by co-targeting antioxidant recovery, metabolic adaptation and organelle-level damage buffering.	(42, 71)

### 5.1 Early ARSI treatment: a pro-ferroptotic vulnerability window (interceptable)

In the early phase of treatment with ARSIs, rapid suppression of AR signaling transiently lowers the ferroptosis threshold in CRPC cells, thereby creating a pro-ferroptotic vulnerability window. This is not merely a time point, but a biological state in which AR inhibition has already disrupted the pre-existing metabolic–antioxidant balance, while compensatory restoration has not yet been completed. The early vulnerability phase is primarily supported by lipidomic and ferroptosis-sensitivity analyses in AR-positive enzalutamide-treated CRPC models. Androgen-targeted therapy induces a quiescent-like state characterized by reduced cell proliferation, ATP production, and mitochondrial activity (42). At the same time, therapy-induced lipid uptake and membrane lipid remodeling may transiently increase sensitivity to GPX4 inhibition, placing cells in a transitional state that is more combustible, yet not fully stabilized. Relevant studies have shown that enzalutamide increases intracellular arachidonic acid levels and the abundance of phosphatidylethanolamines containing polyunsaturated fatty acids. Dynamic lipidomic changes in prostate cancer cells during 21 days of Enz treatment include: first, marked suppression of *de novo* lipid synthesis together with a global increase in exogenous lipid uptake throughout the treatment course; second, significant accumulation of membrane phospholipids accompanied by sustained depletion of storage lipids; and third, a shift of membrane phospholipids toward longer-chain and more unsaturated species, with continuous enrichment of PUFAs. In parallel, ferroptosis sensitivity also changes dynamically during the same 21-day period, showing an early increase at days 4–8, reaching a markedly hypersensitive state by day 14, and remaining highly sensitive at day 21 (42). These observations suggest that days 14–21 may represent an optimal window for early intervention.

Multiple AR-positive prostate cancer cell lines show increased dependence on GPX4 during the early phase after androgen-targeted therapy (68, 69), manifested by markedly enhanced sensitivity to the GPX4 inhibitor RSL3 (42). Notably, when Enz is combined with non-toxic doses of lipase activity inhibitors (orlistat or CAY10499) or the fatty acid δ6 desaturase inhibitor SC26196, the RSL3-induced ferroptosis hypersensitivity, that is, GPX4 dependence, in LNCaP and C4-2B cells is significantly attenuated (42). This suggests that increased lipase activity and fatty acid δ6 desaturase activity may serve as decisive factors that further push cells toward the ferroptosis threshold during this phase.

One unresolved issue is the timing at which the AR-V-driven transcriptional activation of SLC7A11 emerges after Enz treatment and begins to support the subsequent persistent compensatory restoration process (11). It remains unclear at which stage of the dynamic ferroptosis-sensitivity curve this occurs—whether it is already present once marked hypersensitivity is reached, or whether it develops only later, as resistance gradually emerges by day 21. Although days 14–21 appear to represent the period of maximal ferroptosis sensitivity, the molecular features defining this vulnerability window remain incompletely understood. Based on current evidence, this state may reflect a phase in which therapy-induced lipid remodeling and GPX4 dependence have already been established, whereas AR-V-mediated restoration of the SLC7A11/GSH axis has not yet been fully consolidated. However, direct longitudinal measurements of AR-V expression, SLC7A11 abundance, and GSH dynamics during this interval are currently lacking.

Importantly, another study showed that treatment with 10 μmol/L enzalutamide for 4 or 24 hours significantly upregulated SLC7A11 mRNA expression (70). This phenomenon appears more consistent with a short-term aberrant transcriptional response and, given that ferroptosis sensitivity continues to rise in the later phase, should not be equated with the more stable subsequent restoration mediated by AR-Vs.Defining the precise stage at which sustained SLC7A11 expression emerges may provide more accurate guidance for distinguishing early versus late ARSI-based therapeutic strategies in the clinic and represents a promising direction for future research.

### 5.2 Steady-state reconstruction: formation of an anti-ferroptotic resistant state (delayable and destabilizable steady state)

Closure of the vulnerability window does not mean that cells “suddenly become resistant”; rather, it reflects an AR-driven reconstruction process. AR-V can occupy regulatory regions of SLC7A11 and enhance its expression, thereby restoring GSH supply. More recent work further suggests that, in CRPC models, ENZ can also stabilize SLC7A11 protein through a NEDD4L-related mechanism, thereby improving the “refill efficiency” of system Xc^-^ (11, 23). Moreover, combined treatment with NEO2734 and enzalutamide more potently suppresses tumor growth, more strongly inhibits SLC7A11 expression, and increases the lipid peroxidation marker 4-HNE. These data indicate that NEO2734, a dual CBP/p300 and BET inhibitor, can suppress AR variant expression both *in vitro* and *in vivo* and overcome AR-variant-mediated resistance to enzalutamide and ferroptosis in prostate cancer (11).

Once resistance enters the phase of steady-state consolidation, the problem is no longer that a single defense pathway is too strong, but rather that multiple modules begin to provide coordinated buffering: the defense axis is replenished more rapidly, the substrate axis becomes less combustible, the redox axis gains greater buffering capacity, and organelle cooperation becomes more effective at confining damage within a sublethal range. Taken together, single-node pro-ferroptotic strategies at this stage are more likely to produce only short-lived responses followed by rapid compensatory restoration. The central feature of this phase is the establishment of a stable cooperative regulatory network between the AR signaling pathway and ferroptosis-escape pathways, together with the sustained activation of non-AR-dependent ferroptosis resistance mechanisms. Under these conditions, treatment with a single ARSI or ferroptosis inducer is unlikely to achieve durable efficacy, and combination strategies are required to dismantle the resistant steady state.For example,NPC1 and NPC2 are jointly essential for the transport of exogenous cholesterol from lysosomes to the cytoplasm, and both are markedly upregulated in localized and metastatic prostate cancer (42, 71). Studies have shown that prostate cancer cells in an enzalutamide-tolerant state become more sensitive to U18666A. Inhibition of NPC1-mediated cholesterol uptake by U18666A, an inhibitor of the lysosomal NPC1 protein, may therefore represent an effective strategy to exploit the increased dependence of prostate cancer cells on exogenous cholesterol following androgen-targeted therapy (71). However, current evidence is derived predominantly from AR-positive and enzalutamide-tolerant prostate cancer models, and direct comparisons between AR-high and AR-low/AR-negative states are currently lacking. Therefore, whether sensitivity to NPC1/2 inhibition is intrinsically dependent on AR activity remains unclear. Nevertheless, given the well-established role of AR signaling in driving lipid and cholesterol metabolic reprogramming, it is plausible that AR-active CRPC cells may exhibit greater dependence on NPC1/2-mediated cholesterol trafficking, a hypothesis that warrants further investigation.

## 6 Stage-specific intervention strategies: from “uniform drug intensification” to “state-directed disruption”

If the temporal model outlined above is translated into an experimental or therapeutic framework, it yields two fundamentally distinct intervention windows: early interception and late-state dismantling. The goal of the former is to push cells across the ferroptosis threshold while AR dependence still predominates and before metabolic support and anti-ferroptotic defenses have been fully replenished. The latter, by contrast, recognizes that a resistant architecture has already formed and therefore requires hierarchical identification of the dominant module—whether the defense axis, substrate axis, redox axis, or mitochondrial buffering axis—followed by targeted dismantling, rather than blindly repeating the addition of “another ARSI” or “another FIN.”Early interception prioritizes timing, not simply giving more drugs. Its logic is that, after ARSI initiation but before compensatory restoration is complete, decompensatory interventions should be layered in first—for example, weakening system Xc^-^/GSH replenishment, blocking substrate remodeling, or restricting redox regeneration—so as to force cells across the threshold. This means that what truly requires clinical optimization is not merely whether to combine therapies, but when to combine them, for how long, and how to determine whether the vulnerability window remains open.Late-state dismantling, in contrast, prioritizes stratification, because at this stage what appears to be the “same CRPC” often consists of fundamentally different system states. The CARD study has already suggested at the level of clinical outcomes that, in patients previously exposed to ARSI and progressing rapidly, simply switching to another ARSI is less effective than changing to a therapy with a different mechanism, such as cabazitaxel; in essence, this argues against applying the same layer of pressure repeatedly once a resistant state has already consolidated (72). Translated into the AR–ferroptosis framework, this means that in the late phase one should not continue stacking pro-oxidant or pro-ferroptotic agents indiscriminately, but should first determine whether the tumor is better characterized as defense-dominant, substrate-limited, redox-replenished, or mitochondrial-buffered, and then decide whether to dismantle the defense system first, rewire substrate availability first, or disrupt organelle cooperation first and so on. To further enhance the translational relevance of the ferroptosis-threshold framework, we suggest several candidate biomarkers corresponding to its major regulatory modules. For the defense module, SLC7A11, GPX4, FSP1, and AR-V7 may serve as potential readouts; for substrate remodeling, ACSL4, MBOAT2, PUFA-containing phospholipids, and lipid droplet accumulation; for redox buffering, the GSH/GSSG ratio, NADPH availability, and ROS levels; for iron regulation, ferritin, NCOA4, TfR1, and the labile iron pool; and for organelle-network dynamics, DHODH activity, mitochondrial morphology, and MERCS-associated signatures. Dynamic monitoring of these parameters may help determine whether tumors remain within the vulnerability window, are undergoing adaptive reconstruction, or have entered a resistant steady state, thereby facilitating patient stratification and stage-specific therapeutic decision-making.

In practice, dynamic monitoring can be divided into four nodes: baseline stratification (to define AR dependence and resistance risk), early reassessment after ARSI initiation (to determine whether vulnerability signals emerge), intermediate reassessment (to identify trends of SLC7A11/redox replenishment), and re-stratification at progression (to determine whether the tumor has entered a consolidated steady state that requires a change in strategy). Most importantly, the key is not any single static biomarker, but rather the directionality of biomarker change. For example, changes in AR-related resistance indicators may include (**Table 3**): first, persistent AR activation itself, such as AR-V7, AR amplification/copy-number gain, or mutations in the AR ligand-binding domain; these alterations indicate that tumors can maintain or reconstitute AR signaling despite AR inhibition and are generally associated with reduced efficacy of enzalutamide or abiraterone (73–75). Second, bypass compensation may emerge, with glucocorticoid receptor (GR) upregulation being the prototypical example, as GR can partially substitute for AR-driven resistance transcriptional programs (76, 77). Third, intratumoral androgen re-synthesis may be enhanced, for instance through increased HSD3B1 or AKR1C3 expression (78–80). If these changes are further accompanied by RB1/TP53 loss and SOX2-mediated lineage plasticity, this often suggests that the tumor is shifting from AR-dependent resistance toward the more difficult state of AR-independent resistance (81). These indicators do not automatically invalidate pro-ferroptotic strategies. Rather, they indicate that the therapeutic logic must shift from early interception to late-state dismantling. At the same time, this emphasis on the direction of resistance evolution, rather than on single static markers, is consistent with findings from sequential ctDNA studies showing that AR genotypes continue to evolve under ongoing AR-suppressive pressure and drive acquired resistance (82).

**Table 3:** Resistance indicators that redirect ferroptosis-based intervention in CRPC.

Category	Representative changes	Interpretation	Therapeutic implication	References
Persistent AR reactivation	AR-V7, AR amplification/copy-number gain, AR ligand-binding domain mutations	Sustained or restored AR signalling despite AR inhibition	Favors combined strategies targeting persistent AR-dependent rebuilding	(73–75)
Bypass compensation	GR upregulation	Partial substitution for AR-driven resistance programmes	Supports combining ferroptosis-based therapy with blockade of compensatory rescue pathways	(76, 77)
Intratumoral androgen re-synthesis	Increased HSD3B1 or AKR1C3 expression	Reactivation of AR signalling through intracrine androgen production	Supports integrating ferroptosis-oriented therapy with suppression of steroidogenic replenishment	(78–80)
Lineage plasticity transition	RB1/TP53 loss with SOX2-mediated lineage plasticity	Shift from AR-dependent to AR-independent resistance	Indicates a shift from early interception to late-state dismantling	(81)

## 7 Limitations of the model

Although the present framework integrates current evidence into a unified model in which AR signaling functions as a master orchestrator of ferroptosis escape in CRPC, several important limitations should be acknowledged. First, many of the proposed regulatory relationships have been derived from independent studies performed in different experimental systems. Although available evidence supports a central role for AR signaling in coordinating antioxidant defense, lipid remodeling, iron homeostasis, and mitochondrial adaptation, direct experimental validation of these mechanisms operating simultaneously within the same biological context remains limited. Second, the temporal dynamics of ferroptosis escape remain incompletely defined. In particular, the precise timing at which AR-V-driven restoration of the SLC7A11/GSH axis emerges during ARSI treatment remains unclear. While therapy-induced lipid remodeling and ferroptosis hypersensitivity have been characterized during the first weeks of enzalutamide exposure,longitudinal studies tracking AR-V expression, SLC7A11 abundance, and redox adaptation in parallel are currently lacking. Third, the iron-regulatory modules discussed in this review, including the LTF, STAMP2/STEAP4, and NCOA4 pathways, have not yet been systematically evaluated within defined CRPC molecular subtypes.Whether these pathways are coordinately activated, functionally complementary, or context-dependent remains unresolved. Fourth, the proposed role of mitochondria-associated endoplasmic reticulum contact sites (MERCS) as integration hubs linking lipid metabolism,redox signaling, and ferroptosis susceptibility is currently supported primarily by indirect evidence. Direct spatial and functional characterization of MERCS remodeling in CRPC remains limited. Fifth, the generalizability of this framework is constrained by the heterogeneity of CRPC. The central thesis is most applicable to AR-dependent or AR-plastic CRPC and should not be extrapolated to AR-low or AR-independent tumors, including those undergoing neuroendocrine-like transformation. In such tumors, survival may no longer rely primarily on the AR axis, and the factors determining ferroptosis sensitivity are likely shifted to other dominant regulatory programs (83, 84). Although this framework is most relevant to AR-dependent and AR-plastic CRPC, ferroptosis regulation may still be influenced by prior AR activity in lineage-divergent states. Key metabolic, lipid-remodeling, redox, and mitochondrial programs established under sustained AR signaling can persist even after partial loss of AR dependence. During progression, the dominant organizer of cellular state may shift gradually from AR toward lineage-specific drivers such as SOX2, EZH2, or MYCN, following a continuum rather than an abrupt switch (85, 86). Therefore, the ferroptosis-threshold framework may remain applicable in lineage-plastic CRPC, as its core regulatory modules likely continue to operate under reprogrammed upstream control. Direct experimental validation in SOX2-, EZH2-, or MYCN-driven contexts is lacking, and this interpretation should be regarded as a conceptual hypothesis for future investigation. Finally, the concept of a “ferroptosis threshold” used throughout this framework should be viewed as a theoretical construct rather than a directly measurable biological parameter. At present, no standardized quantitative method exists to define or monitor ferroptosis thresholds in clinical samples. Future studies integrating lipidomics, metabolomics, redox profiling, and single-cell analyses may help establish quantitative biomarkers capable of operationalizing this concept.Taken together, these limitations do not diminish the value of the proposed framework but rather highlight key areas where further mechanistic and translational investigations are required. Future efforts aimed at validating the temporal, spatial, and quantitative dimensions of ferroptosis regulation may further refine our understanding of AR-driven ferroptosis escape and facilitate clinical translation.

## 8 Conclusions and perspectives

The principal contribution of this review is therefore to propose an explanatory framework that is more consistent with the evolutionary logic of CRPC: in most AR-dependent CRPCs, AR signaling acts first as the primary organizing axis, coordinating across multiple layers the supply of defense systems, membrane lipid substrates, redox buffering, and the iron toxicity boundary; ferroptotic stress, by contrast, functions mainly as a feedback modulator that reshapes this organizing axis at different stages, rather than serving intrinsically as the initiating dominant force. Future research will require three major advances. First, dynamic biomarkers, together with ctDNA- and multi-omics-based monitoring approaches, should be developed to identify transitions from vulnerability-window opening to window closure and ultimately to steady-state consolidation. Such efforts may also help establish molecular classifiers based on AR activity, AR-V expression, ferroptosis-related signatures, and metabolic states, thereby enabling more precise patient stratification. Second, clinical trial design should move beyond the assumption that all CRPCs share a common therapeutic vulnerability. Instead, stratified and adaptive strategies should be adopted according to AR status, lineage state, metabolic dependencies, and ferroptosis susceptibility. For example, therapeutic vulnerabilities associated with cholesterol trafficking, NPC1/2-mediated lipid transport, ferroptosis sensitivity, or AR-dependent metabolic reprogramming may differ substantially between AR-active and AR-independent disease states. Third, mechanistic endpoints should be incorporated alongside conventional clinical endpoints. Dynamic state panels integrating lipid remodeling, redox adaptation, iron metabolism, and AR signaling activity may provide a means to directly evaluate whether disruption of the AR–ferroptosis state can be translated into durable therapeutic responses and improved survival outcomes. Ultimately, validating the temporal, spatial, and quantitative dimensions of ferroptosis regulation will be essential for transforming the current conceptual framework into a clinically actionable model. Such efforts may facilitate the development of state-guided therapeutic strategies capable of exploiting ferroptosis vulnerabilities throughout the evolutionary trajectory of CRPC.
